# Method of straw ditch-buried returning, development of supporting machine and analysis of influencing factors

**DOI:** 10.3389/fpls.2022.967838

**Published:** 2022-09-15

**Authors:** Han Tang, Changsu Xu, Wenlong Xu, Yanan Xu, Yushun Xiang, Jinwu Wang

**Affiliations:** College of Engineering, Northeast Agricultural University, Harbin, China

**Keywords:** conservation tillage, straw returning, agronomy, soil disturbance amount, chain ditcher

## Abstract

This paper aims to solve the problems of the low quality and shallow depth of the traditional straw return method. According to the requirements of the new furrow burial and return agronomic model, a corn straw ditch-buried returning machine was designed that could simultaneously complete the processes of picking, conveying, ditching, soil-covering and pressing. Key components were theoretically analyzed and designed, such as the pickup device, ditching device and straw-guiding soil-covering and pressing device. Based on a field experiment, the main factors influencing the effects of straw picking, soil ditching and straw return were studied. Both forward speed and pickup device speed significantly affected the straw picking rate. The ditching area, ditching width consistency factor and ditching depth stability factor gradually decreased with increasing forward speed and gradually increased with increasing trenching device speed. There was a significant interaction among the forward speed, pickup device speed and ditching device speed. At a forward speed of 1.68 m/s, the picking device speed was 330 r/min, the ditching device speed was 290 r/min, and the highest straw return rate was 93.65%.

## Introduction

Conservation tillage technology is a kind of green agricultural farming technology with mechanization as the means and no-till mulching and soil and water conservation as the core. Conservation tillage technology can effectively reduce wind and water soil erosion and improve the soil moisture storage capacity by covering the ground with crop straw and stubble and sowing *via* no-till or less-till methods (Huang W. et al., 2021; Qi et al., 2021; Zhang et al., 2021). In 2021, China’s corn planting area reached 4.3 × 10^7^ hectares with a straw amount of 2.3 × 10^8^ tons, which accounted for approximately 17% of the global corn straw. Corn straw production is high and difficult to manage. How to employ conservation tillage technology to manage straw remains a key issue to be solved (Li et al., 2021; He et al., 2022).

There are five main types of comprehensive straw utilization options: fertilization, fuel, raw material, feed, and base material options (Wang J. et al., 2017). As a straw fertilizer utilization method, mechanized straw return in the field is the most direct form of comprehensive utilization of straw resources (Tang et al., 2020). At present, the main method of mechanized straw return is to evenly crush and spread straw in the field after crop harvesting and return straw to the field by rototilling or plowing (He et al., 2018). Since the secondary crushing and burying process is tedious and affects the economic return, most farmers directly plow the land with a rotary tiller or directly bury and return straw to the field. Wang et al. (2019) proposed a rice straw whole-plant deep burial return technique. This technique effectively solved the problem of shallow straw return depths. Wang R. et al. (2017) developed a straw deep burial, returning and stubble removal machine. This machine performed well in terms of ditching, stubble removal and soil breakage. Lin et al. (2017) and Tian et al. (2018) built a deep burial straw returning machine. This machine was the first to apply a spiral ditching device to achieve deep straw burial and satisfy the agronomic requirements of deep burial and return to the field. In addition, openers are the key components of straw deep burial and return to the field, including spar, disc and chain openers (Gao et al., 2018). The above research has achieved good operational results, but regarding the agronomic requirements of different crop straw types, numerous machine types remain necessary to meet the needs of straw return.

At present, most of the research on the treatment of straw return to the field focuses on the effect of straw return to the field, soil physicochemical properties, subsequent crop growth and yield effects, etc. Liu et al. (2020) investigated the changes in water use efficiency of wheat during different growth periods *via* four tillage methods, including straw return. Zhou et al. (2020) researched the effect of the spatial distribution of straw in soil after straw return *via* different tillage methods. The results of Chen et al. (2017) indicated that straw return to the field increased the thousand grain weight, seed yield, post-flowering dry-matter accumulation rate and nitrogen efficiency of wheat. Akhtar et al. (2018) investigated the effectiveness of straw return to the field in improving the soil organic matter content. Moreover, the straw return method can reduce greenhouse gas emissions (Zhang et al., 2018), increase the enzyme activity in soil (Jin et al., 2009; Huang Y. et al., 2021), improve the structure of soil aggregates, etc. (Benbi and Senapati, 2010; Cates et al., 2016).

Straw furrow burial and return is a new soil tillage technique that organically combines the full amount of straw safely returned to the field and local soil rotation deep plowing. This technique can effectively alleviate the contradiction whereby straw is difficult to return to the field under less no-till conditions. This technique is important to improve soil properties, increase the soil tillage depth, reduce carbon emissions from farmland, and mitigate environmental problems caused by straw burning (Yang et al., 2017, 2020a,b; Li et al., 2020). Straw furrow burial and return require straw collection, ditching, burying, soil covering and other multistep processes. At present, straw is still buried in furrows and returned to the field *via* conventional mechanical devices alone. The low degree of mechanization and complicated operation procedures limit the promotion and development of the straw furrow burial and return technique.

This paper designed a corn straw ditch-buried returning machine that could complete the processes of picking, conveying, ditching, soil covering and pressing simultaneously. By analyzing the agronomic model of furrow burial and return, this paper theoretically designed key components, such as the picking device, chain ditching device and straw-guiding soil-covering and pressing device. Based on field experiments, the main factors that affect the effectiveness of straw picking, soil trenching and straw return were studied.

## Materials and methods

### Agronomic requirements

**Figure 1:**
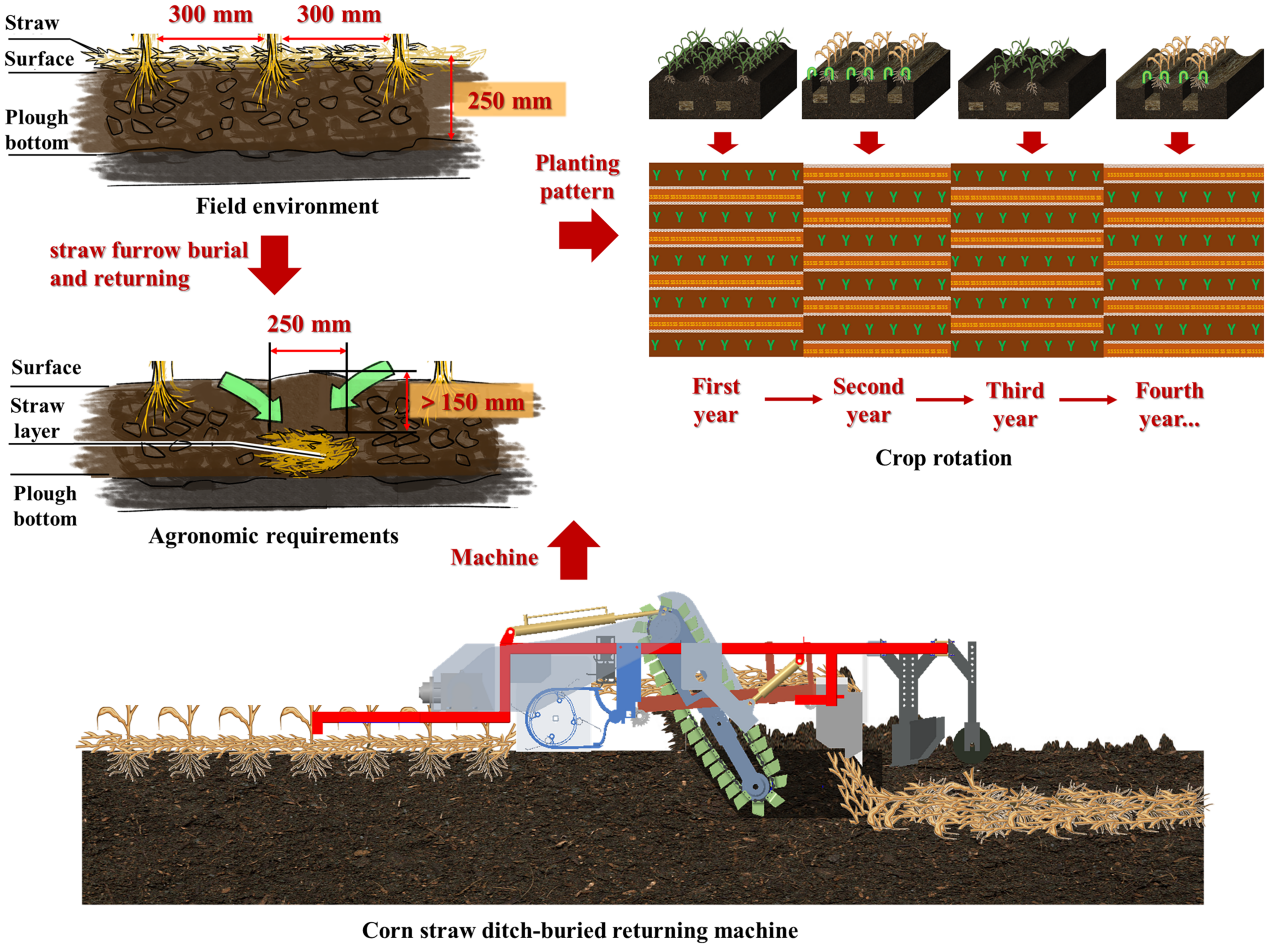
Agronomic requirements of straw ditch-buried returning.

After corn harvesting, crushed stalks were evenly scattered in the field. The residual corn root stubble rows were generally spaced at 300 mm. A strip ditch with a width of 250 mm and a depth of 300 mm was opened between the root stubble rows and the ditching device of the straw burial and returning machine. The pickup device collected straw within the working width toward the opened ditch and covered the soil to ensure deep straw burial at 150–300 mm from the surface. In the next year, straw was again deeply buried in corn stubble rows, and corn was sown between the rows buried in the previous year. This process was repeated in a rotational manner. The agronomic requirements of furrow burial and return to the field are shown in Figure 1.

This method can effectively break the plow bottom and realize the function of deep loosening at regular intervals. Moreover, the organic matter deficiency in the 150–300 mm soil layer is high. Straw decomposition in soil after deep burial can improve the soil structure, increase the soil porosity, form more humus, realize soil carbon sequestration, reduce carbon dioxide emission, promote soil organic matter accumulation, and enhance the water and moisture storage capacity (Yang et al., 2016). In addition, straw buried in furrows and returned to the field can concentrate topsoil and straw into strips. This method achieves a satisfactory fertilization effect, fertilizes the subsurface layer, expands the tillage layer, and improves soil organic matter.

### Structure and working principle of the machine

**Figure 2:**
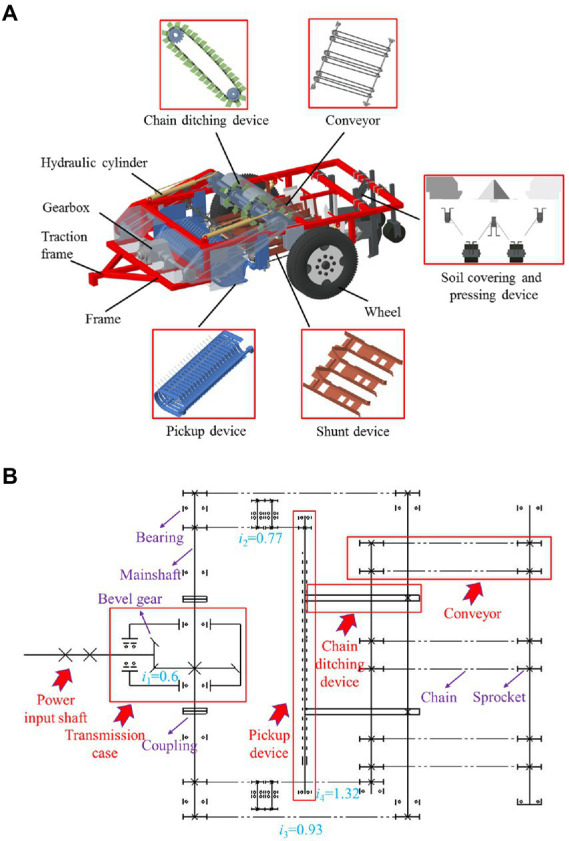
Corn straw ditch-buried returning machine. **(A)** Overall structure; **(B)** transmission route. Where *i*_1_ is the transmission ratio of the bevel gear, *i*_2_ is the transmission ratio of the pickup device, *i*_3_ is the transmission ratio of the chain ditching device, *i*_4_ is the transmission ratio of the conveyor, and the unspecified transmission ratio is 1.

The corn straw ditch-buried returning machine mainly consists of a frame, a pickup device, a diversion device, a conveyor, a chain ditching device, a straw-guiding soil-covering and pressing device and a related transmission device. The entire structure is shown in Figure 2A. This machine can simultaneously complete picking, conveying, ditching, soil-covering and pressing. Among these devices, the pickup device is configured at the front of the entire machine to effectively collect corn straw and smoothly discharge the collected straw onto the conveyor to avoid returning the straw. The chain ditching device can flexibly rotate to cut and throw soil onto both sides of the ditch. While ensuring stable ditching, soil return can be effectively avoided. The straw-guiding soil-covering and pressing device discharges corn straw, which is conveyed by the conveyor, into the ditch, subsequently directs the soil on both sides of the ditch atop the straw, and presses the whole mass together. The device compacts the unconsolidated straw and soil twice to create a good field environment for subsequent operation sessions. The technical parameters of the corn straw ditch-buried returning machine are provided in Table 1.

**Table 1:** Technical parameters of the corn straw ditch-buried returning machine.

Parameters	Value	Unit
Matching power	≥90	kW
Dimension (length× width× height)	5,200 × 2,990 × 1,420	mm
Efficiency	0.5–2	m/s
Weight	2,500	kg
Working width	1,600	mm
Ditching width	250	mm
Ditching depth	300	mm
Pickup rate	≥85	%
Returning rate	≥85	%

The power is input into the bevel gear of the transmission case by the power input shaft, which converts the power from the forward direction to the horizontal direction and realizes speed change. In the horizontal direction of the machine, two symmetrical main shafts are arranged. The main shaft and transmission case are connected by couplings and equipped with sprockets. The power is transmitted to the pickup device, conveyor and ditching device through sprockets with different transmission ratios. The transmission route is shown in Figure 2B.

During operation, the corn straw ditch-buried returning machine is connected to the tractor through the traction frame to transmit power. Corn straw in the field is discharged backward by the pickup device. The diversion device diverts straw toward the conveyor. The straw is smoothly transported on the conveyor to the ditch, which has been created by the ditching device. The straw-guiding soil-covering and pressing device directs the soil on both sides of the ditch toward the top of the straw and conducts flattening and dense pressing operations. These steps constitute a complete working process.

### Design of the key components

The key components of the corn straw ditch-buried returning machine are the pickup device, chain ditching device and straw-guiding soil-covering and pressing device. The quality of corn straw that is buried in furrows and returned to the field directly determines the working quality of these key components. Therefore, the parameters that affect the working quality of each key component were studied and designed. The parameters of each key component are listed in Table 2.

**Table 2:** Key component parameters.

Parameter	Value	Unit	
Pickup device	Rotational speed	≤ 350	r/min
	Pickup turning radius	370	mm
	Number of pickup spring teeth	4	/
	Ground clearance	100	mm
Chain ditching device	Chain gear speed	≤ 400	r/min
	Chain line speed	≤ 1.5	m/s
	Ditching knife height	220	mm
	Ditching knife width	125	mm
	Ditching knife pitch	130	mm
Straw-guiding soil-covering and pressing device	Angle between the straw-guiding mechanism and horizontal plane	60	°
	Angle between the soil-covering mechanism and forward direction	45	°
	Diameter of the pressing mechanism	400	mm

#### Design of the pickup device

**Figure 3:**
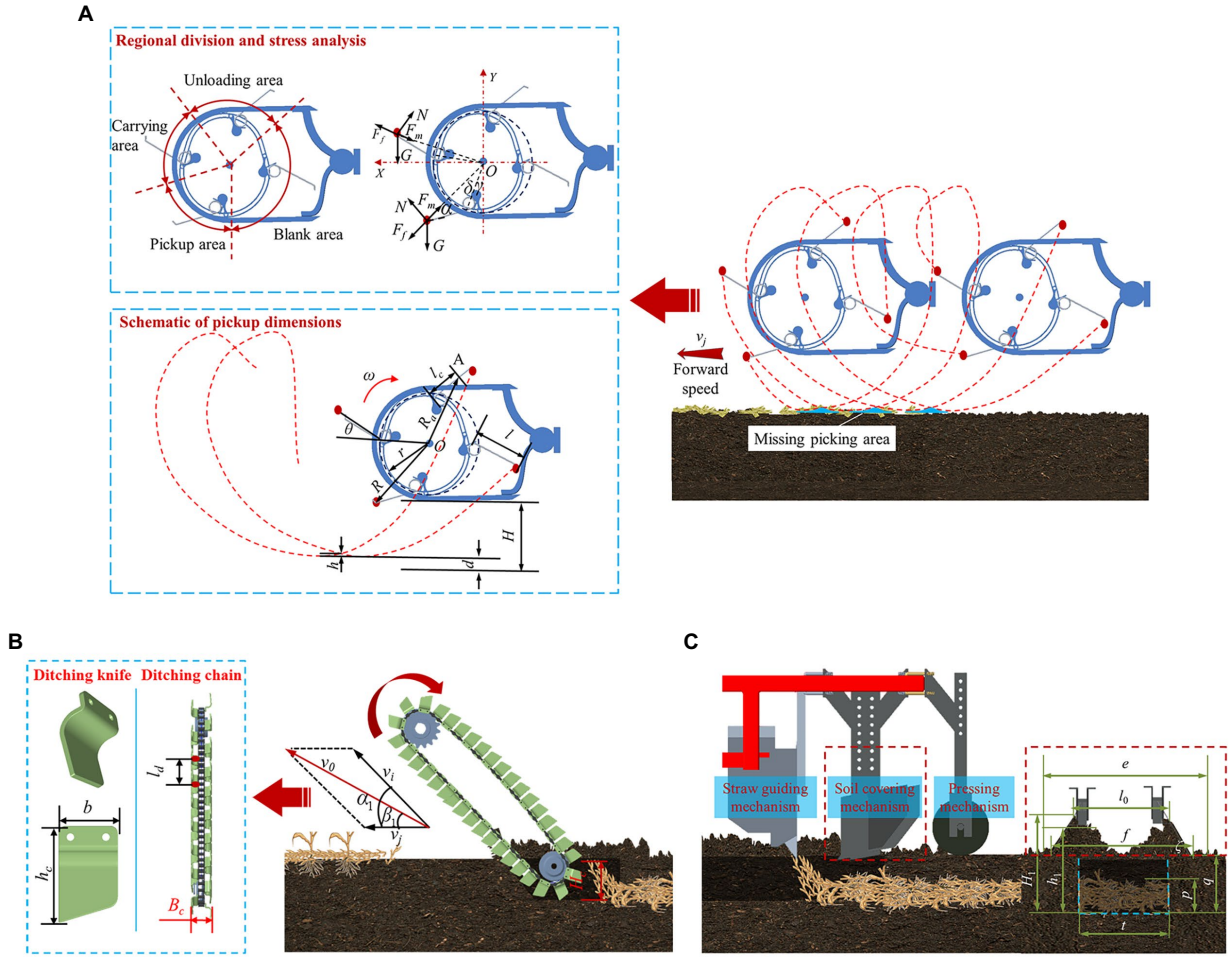
Design of the key components of the corn straw ditch-buried returning machine. **(A)** Design diagram of the pickup device; **(B)** design diagram of the chain ditching device; **(C)** design diagram of the guiding soil-covering and pressing device.

The pickup quality is directly determined by the trajectory of the pickup spring teeth of the pickup device. The trajectory of the pickup teeth movement exhibits a pendular shape. The missing picking area occurs at the intersection of two adjacent pendular trajectories. A design diagram of the pickup device is shown in Figure 3A. To avoid missing picking areas, the following conditions must be satisfied:


(1) h≤H−d

(2) $$\{\begin{array}{l} \lambda =v/v_{j}=\left(\beta -\varphi \right)/2\sin\varphi /2\\ \varphi =2\arccos\left(1-h/R\right) \end{array}$$

where *H* is the pickup rack height from the ground, mm; *h* is the height of the missing picking area, mm; *d* is the ground clearance of the spring teeth, mm; λ is the ratio of the linear speed at the end of the spring teeth to the forward speed of the machine; v is the linear speed at the end of the spring teeth, m/s; $v_{j}$ is the machine forward speed, m/s; β is the adjacent teeth rod angle, °; φ is the drum angle corresponding to *h*, °; *R* is the turning radius of the end point of the spring teeth relative to point *O*, mm.

In this study, the four spring tooth shafts were evenly distributed along the circumference of the drum, and β was 90°. According to the thickness of straw laid in the field after corn harvesting and to prevent the contact between bullet teeth and ground from damaging the structure, the initial setting *d* was 20 mm, and *H* was set to 120 mm, so *h* ≤ 100 mm.

When the spring teeth were located at the top of the unloading area, the horizontal velocity of midpoint A extending from the pickup rack section should reach 0 as follows:


(3) $$v_{j}+\omega R_{a}\sin\omega t=0$$

where ω is the angular velocity of the spring teeth end, rad/s, and *R_a_* is the turning radius of point A relative to point *O*, mm.

Substituting relevant structural parameters into Equation (3), we obtain:


(4) $$\{\begin{array}{l} v_{j}=-\omega R_{a}\sin\omega t=\sqrt{r^{2}+l_{c}+2rl_{c}\cos\theta }\\ \begin{array}{c} \lambda =v/v_{j}=\omega R/v_{j}\\ =\sqrt{\left(r^{2}+l^{2}+2rl\cos\theta \right)/\left(r^{2}+l_{c}^{2}+2rl_{c}\cos\theta \right)} \end{array}\\ R_{a}=\sqrt{r^{2}+l_{c}^{2}+2rl_{c}\cos\theta }\\ R=\sqrt{r^{2}+l^{2}+2rl\cos\theta } \end{array}$$

where *r* is the rotation radius of the spring tooth roller, mm; *l_c_* is the distance between the middle point of the spring tooth that extends from the pickup frame and the spring tooth roller, mm; *l* is the length of the spring tooth, mm; θ is the angle between the line from the spring tooth roller to the center of rotation and the spring tooth, °.

According to the agronomic requirements of straw return, the maximum forward speed of the machine was set to 2 m/s. The rotation radius of the spring tooth roller is a quantitative parameter. According to the size of the entire machine, *r* was set to 220 mm, *l* was set to 220 mm, and *R* was set to 370 mm. The above quantification process was realized. A reverse calculation of the parameters determined that the included angle θ between the line from the spring tooth roller to the center of rotation and the spring tooth was 49°.

The pickup device can be divided into four areas for work trip completion: pickup area, carrying area, unloading area and blank area. Among these areas, no pileup or slip in the pickup area and no ejection into the carrying area are necessary to effectively unload straw. The factors that affect smooth straw collection for transport were explored.

First, the forces acting on straw in the pickup area were analyzed. These forces can be projected onto the *X* and *Y* axes as follows:


(5) $$\{\begin{array}{l} \begin{array}{l} F_{m}\sin\left(\gamma +\delta \right)-F_{f}\sin\left(\alpha +\gamma +\delta \right)\\ -N\cos\left(\alpha +\gamma +\delta \right)=0 \end{array}\\ \begin{array}{l} F_{m}\cos\left(\gamma +\delta \right)+N\sin\left(\alpha +\gamma +\delta \right)\\ -F_{f}\cos\left(\alpha +\gamma +\delta \right)-mg=0 \end{array} \end{array}$$

where *F_m_* is the centripetal force acting on straw, *N*; *F_f_* is the frictional force acting on straw, *N*; *N* is the support force acting on straw, *N*; α is the angle between the connecting line from the straw pickup centroid to the rotation center and the spring tooth, °; γ is the angle between the line from the center of rotation to the spring tooth roller and the vertical direction, °; δ is the angle between the line from the center of rotation to the spring tooth roller and the line from the center of rotation to the straw pickup centroid, °; *m* is the weight of the collected straw, kg; *g* is the gravitational acceleration (9.8 N/kg).

Then, the following is obtained:


(6) $$\{\begin{array}{l} F_{m}=m\omega^{2}R_{a}\\ N=mg\sin\left(\alpha +\gamma +\delta \right)\\ F_{f}=N\mu =mg\mu \sin\left(\alpha +\gamma +\delta \right) \end{array}$$

Therefore, the conditions for straw not to pile up are as follows:


(7) $$\{\begin{array}{l} \begin{array}{l} F_{m}\geq \left[F_{f}\sin\left(\alpha +\gamma +\delta \right)+N\cos\left(\alpha +\gamma +\delta \right)\right]\\ /\sin\left(\gamma +\delta \right) \end{array}\\ \omega \geq \sqrt{\begin{array}{l} \left[2g\mu \sin^{2}\left(\alpha +\gamma +\delta \right)+g\sin2\left(\alpha +\gamma +\delta \right)\right]\\ /2R_{a}\sin\left(\gamma +\delta \right) \end{array}} \end{array}$$

The conditions for straw not to experience slip are as follows:


(8) $$\{\begin{array}{l} \begin{array}{l} F_{m}\geq \left[F_{f}\cos\left(\alpha +\gamma +\delta \right)+mg-N\sin\left(\alpha +\gamma +\delta \right)\right]\\ /\cos\left(\gamma +\delta \right) \end{array}\\ \omega \geq \sqrt{\begin{array}{l} \left[g\mu \sin2\left(\alpha +\gamma +\delta \right)+2g-2g\sin^{2}\left(\alpha +\gamma +\delta \right)\right]\\ /2R_{a}\cos\left(\gamma +\delta \right) \end{array}} \end{array}$$

The stress acting on straw in the carrying area was analyzed. *Via* force projection onto the spring teeth, the following force relationship can be obtained:


(9) $$\{\begin{array}{l} mg\cos\left(\pi -\gamma \right)+F_{m}-F_{f}=0\\ N=mg\sin\left(\pi -\gamma \right)\\ F_{m}=m\omega^{2}R_{a}\\ F_{f}=N\mu =mg\mu \sin\left(\pi -\gamma \right) \end{array}$$

Then, the conditions for straw not to be ejected into the carrying area are as follows:


(10) $$\{\begin{array}{l} F_{m}\leq F_{f}-mg\cos\left(\pi -\gamma \right)\\ \omega \leq \sqrt{\left[g\mu \sin\left(\pi -\gamma \right)-g\cos\left(\pi -\gamma \right)\right]/R_{a}} \end{array}$$

In summary, 15.71 rad/s ≤ω≤ 28.27 rad/s.

#### Design of the chain ditching device

The chain ditching device can stably and efficiently open the ditch and discharge soil onto both sides of the ditch. The quality of ditching directly impacts the quality of straw covering. A design diagram of the chain ditching device is shown in Figure 3B.

The efficiency of the chain ditching device can be determined as:


(11) $$\eta =3.6\times 10^{-3}H_{c}B_{c}v_{j}$$

where η is the work efficiency of the chain ditching device, m^3^/h; $H_{c}$ is the depth of ditching, mm; $B_{c}$ is the width of ditching, mm.


(12) $$v_{0}=\sqrt{v_{j}^{2}+v_{i}^{2}+2v_{j}v_{i}\cos\alpha_{1}}$$

where $v_{0}$ is the absolute speed of the chain ditching device, m/s; $v_{i}$ is the line speed of the ditching knife, m/s, set to 1.5 m/s; $\alpha_{1}$ is the angle between the ditching knife and the horizontal plane, °, generally in the range of 48°–65° and initially set to 50°, which results in $v_{0}$ =3.18 m/s.

The angle between the absolute speed of the chain ditching device and the horizontal plane is $\beta_{1}$ , which can be calculated with Equation (13):


(13) $$\tan\beta_{1}=v_{i}\sin\alpha_{1}/\left(v_{i}\cos\alpha_{1}+v_{j}\right)$$

and $\beta_{1}$ = 44^°^.

According to the requirements of the actual working conditions, if the height of the ditching knife increases the load, the body of the ditching knife can be deformed or broken. This paper designed the height of the ditching knife as *h_c_* = 220 mm.

The width of the ditching knife and thickness of the cut soil can be calculated with Equation (14).


(14) a=b/(10~15)

where *a* is the thickness of the soil cut by a set of ditching knives, mm, and initially set to 9 mm; *b* is the width of the ditching knife, mm, and set to 125 mm.

When the preliminary design of the ditching knife was finished, the transportation capacity of the chain ditching device and soil clearing capacity were evaluated. The productivity of the chain ditching device was calculated based on the discharge capacity $\eta_{0}$ as follows (Liu et al., 2012):


(15) $$\{\begin{array}{l} \begin{array}{l} \eta_{0}=3.6\times 10^{-3}bh_{c}v_{i}\left\{1-l_{d}/\left[2h\tan\left(\alpha_{1}-\varphi_{1}\right)\right]\right\}\\ ,\alpha_{1}\leq \varphi_{1}+\arctan\left(h_{c}/l_{d}\right) \end{array}\\ \begin{array}{l} \eta_{0}=3.6\times 10^{-3}bh_{c}v_{i}\left\{h_{c}/\left[2l_{d}\tan\left(\alpha_{1}-\varphi_{1}\right)\right]\right\}\\ ,\alpha_{1}>\varphi_{1}+\arctan\left(h_{c}/l_{d}\right) \end{array}\\ \begin{array}{l} \eta_{0}=3.6\times 10^{-3}bh_{c}v_{i},\\ \alpha_{1}\leq \varphi_{1} \end{array} \end{array}$$

where $\varphi_{1}$ is the natural resting angle of loose soil, °. Due to the sticky and heavy soil after corn harvesting, this angle was set to 35°.

It was determined whether substituting each parameter into Equation (15) could satisfy $\alpha_{1}>\varphi_{1}+\arctan h_{c}/l_{d}$ . Then, $\eta_{0}$ =625.26 m^3^/h was obtained.

After calculation, η and $\eta_{0}$ were compared to ensure that Equation (16) was satisfied to be consistent with the design requirements.


(16) $$\eta \leq \eta_{0}/k_{d}\lambda$$

where $k_{d}$ is the soil loosening factor and chosen to be 1.01, and λ is the dispersion coefficient related to the chain movement speed and chosen to be 0.90.

The chain ditching device is mainly affected by the cutting resistance during operation. Then, the total cutting resistance *F_k_* is calculated with Equation (17).


(17) $$\{\begin{array}{l} F_{t}=I_{tu}a\left(bk_{j}+z_{c}k_{s}\right)\left(1+0.256\mathrm{lg}v_{i}\right)k_{y}\\ z_{t}=H_{c}/\sin\left(\alpha_{1}l_{d}\right)\\ F_{k}=F_{t}z_{t} \end{array}$$

where $I_{tu}$ is the impact value of the firmness meter, selected as 11; *k_j_* is the coefficient of the difficulty in soil processing, selected as 4.3 × 10^4^ Pa; *k_s_* is the proportion coefficient of the side knife cutting soil, selected as 1.8 × 10^3^ N/m; *z_c_* is the cutting coefficient of the closed side tool, selected as 2; *k_y_* is the influence coefficient of the cutting angle, selected as 0.9; *F_t_* is the resistance of each ditching knife to soil cutting, N; *z_t_* is the total number of ditching knives in the chain ditching device, selected as 38; *F_k_* is the total cutting resistance of the chain ditching device, N.

#### Design of the straw-guiding soil-covering and pressing device

The straw-guiding soil-covering and pressing device is located behind the chain ditching device. The straw-guiding soil-covering and pressing device mainly consists of a straw-guiding mechanism, a soil-covering mechanism and a pressing mechanism. The straw-guiding mechanism can accurately guide straw on the conveyor into the ditch *via* gravity. The soil-covering mechanism pushes soil on both sides of the ditch toward the top of the straw occurring in the ditch, and the pressing device subsequently compacts the soil. A structure diagram is shown in Figure 3C. The following analysis focuses on the relationship among the parameters of the soil-covering mechanism. It was assumed that the soil in the ditch within the same section exhibited equal surface area to the soil on both sides of the ditch, as follows:


(18) $$qt=eh_{1}/2$$

where *q* is the depth of ditching, mm, chosen as 300 mm; *t* is the width of ditching, chosen as 250 mm; *h*_1_ is the distance between the top of the soil on both sides of the ditch and the bottom of the ditch after ditching, mm; *e* is the distance between the soil edges on both sides of the ditch (the value omits the width of the ditch), mm.

To ensure the burying effect of straw, the amount of soil covered by the soil-covering mechanism should be larger than the amount of soil required to cover the straw layer.


(19) $$\frac{1}{2}f\left(H_{1}-q\right)>t\left(q-p\right)$$

where *f* is the width of the soil-covering mechanism operation (the value omits the width of the ditch), mm, and *H*_1_ is the distance from the top of the soil-covering mechanism to the bottom of the ditch, mm. According to the overall configuration requirements, *H*_1_ was 500 mm. The solution was *f* > 215 mm, and the design value was 240 mm.

Then, the angle ζ between the soil-covering mechanism and the forward direction satisfied the relationship of Equation (20).


(20) $$\tan\zeta =\left(f-l_{0}\right)/\left[2\left(H_{1}-q\right)\right]$$

where *l*_0_ is the top distance of the soil-covering mechanism, mm, chosen as 40 mm (the value omits the width of the ditch). Then, the angle between the soil-covering and forward directions was designed as 45°.

### Field experiment

**Figure 4:**
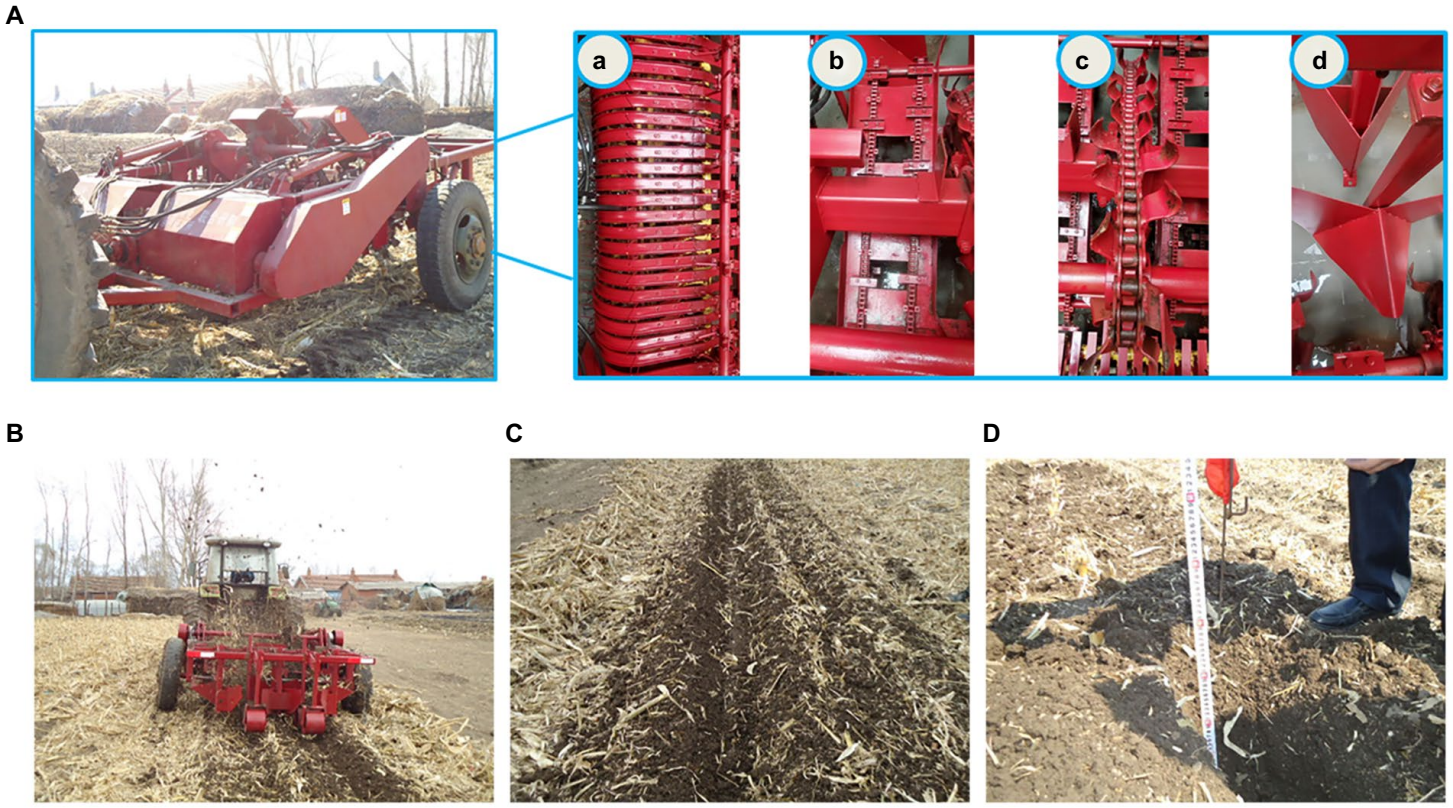
Field experiment. **(A)** Machine; **(B)** working status; **(C)** effect; **(D)** index measurement. (a) indicates the pickup device; (b) indicates the conveyor; (c) indicates the chain ditching device; and (d) indicates the guiding soil-covering and pressing device.

A field experiment was conducted in September 2021 at the experimental site of the Northeast Agricultural University in Acheng District, Harbin city, Heilongjiang Province, as shown in Figure 4. The experiment was conducted in corn fields after the fall combined harvesting operations. The corn stubble height was 6–8 cm, the amount of corn straw was 6,833 kg/hm^2^, and the moisture content in straw was 18.30%. The soil type was black loam, and the soil moisture content was 22.71, 19.83 and 18.97% measured at depths of 0–10 cm, 10–20 cm and 20–30 cm, respectively. The soil compactness was 0.88, 2.67 and 3.73 MPa, respectively. The ditching depth was 300 mm. Supporting power was provided by a Leimu 1304 tractor.

The test method referred to national standards GB/T24675.62009 (conservation tillage machinery straw crushing and returning machine), NY/T740 2003 (field trenching machinery operation quality) and JB/T5160 2010 (forage picker).

In this study, a field performance experiment of the developed corn straw ditch-buried returning machine was conducted in terms of the straw picking rate, furrow opening stability and furrow burial return rate. In the experiment, each group was selected with a 20-m working stroke, each group of indicators was measured three times, and the results were averaged.

The straw picking rate and furrow opening stability constitute intermediate indicators, and the quality of both operations can impact the furrow burial return rate. Therefore, single-factor experiments were separately conducted to study the effect pattern of each factor on these indicators. In the experiments, forward speeds of 0.50, 0.80, 1.10, 1.40, 1.70, and 2.00 m/s at a pickup device rotational speed of 270 r/min and pickup device rotational speeds of 150, 190, 230, 270, 310, and 350 r/min at a forward speed of 1.40 m/s were employed as experimental factors to explore the effect on the straw picking rate. The influence on the stability of ditching was studied considering forward speeds of 0.50, 0.80, 1.10, 1.40, 1.70, and 2.00 m/s at a ditching device rotational speed of 320 r/min and ditching device rotational speeds of 200, 240, 280, 320, 360, and 400 r/min at a forward speed of 1.40 m/s as experimental factors.

The straw return rate was the final indicator. A three-factor and five-level orthogonal rotational combination test design was realized with the forward speed, rotational speed of the pickup device and rotational speed of the ditching device as the experimental factors to study the interaction effects among these factors and the best combination of parameters. The experimental factor level coding is summarized in Table 3.

**Table 3:** Experimental factor level coding.

Code	Forward speed *X*_1_ (m/s)	Pickup device rotational speed *X*_2_ (r/min)	Trenching device speed *X*_3_ (r/min)
−1.68	0.50	150	200
−1	0.80	190	240
0	1.25	250	300
1	1.70	310	360
1.68	2.00	350	400

To further verify and analyze the operation effect of the optimized straw returning machine, the optimized forward speed, rotation speed of the pickup device and rotation speed of the chain ditching device were taken as the test factor level, and the straw returning rate was taken as the test index to perform the field verification test. The test was repeated three times, and the results were averaged and compared with the software optimization results.

### Data processing

#### Straw picking rate

A collection device was installed behind the pickup device. The weight of straw collected within each set of strokes and the weight of residual straw within this set of strokes were measured. The calculation method is expressed as Equation (21).


(21) $$P=M_{0}/\left(M_{0}+M_{1}\right)\times 100\%$$

where *P* is the straw picking rate, %; *M*_0_ is the weight of the straw collected, g; *M*_1_ is the weight of the residual straw, g.

#### Stability of ditching

The stability of ditching was measured based on the amount of soil disturbance, ditching width consistency factor and ditching depth stability factor. In each group of trips, three positions were chosen on average to measure the ditch size. The soil disturbance was calculated using a previously reported method in the literature based on the area of the ditch boundary (Liu et al., 2017). The ditching width consistency factor can be calculated with Equation (22).


(22) $$\{\begin{array}{l} \mu_{a}=\overset{n}{\underset{i=1}{\sum }}w_{i}/n\\ \sigma_{a}=\sqrt{\overset{n}{\underset{i=1}{\sum }}{\left(w_{i}-\mu_{a}\right)}^{2}/\left(n-1\right)}\\ \kappa_{a}=1-\sigma_{a}/\mu_{a}\times 100\% \end{array}$$

where $\mu_{a}$ is the average ditching width, mm; $w_{i}$ is the ditching width value of a single measurement, mm; *n* is the number of measurement positions, chosen as 3 (repeat the measurement three times); $\sigma_{a}$ is the standard deviation of the trenching width, mm; $\kappa_{a}$ is the ditching width consistency factor, %.

The ditching depth stability factor can be determined with Equation (23).


(23) $$\{\begin{array}{l} \mu_{b}=\overset{n}{\underset{i=1}{\sum }}h_{i}/n\\ \sigma_{b}=\sqrt{\overset{n}{\underset{i=1}{\sum }}{\left(h_{i}-\mu_{b}\right)}^{2}/\left(n-1\right)}\\ \kappa_{b}=1-\sigma_{b}/\mu_{b}\times 100\% \end{array}$$

where $\mu_{b}$ is the average ditching depth, mm; $h_{i}$ is the ditching depth value of a single measurement, mm; $\sigma_{b}$ is the standard deviation of the ditching depth, mm; $\kappa_{b}$ is the consistency coefficient of the ditching depth, %.

#### Straw return rate

In each group of trips, the weight of straw in the soil surface layer before ditch burial and the weight of straw in the soil surface layer after ditch burial were separately measured. The straw return rate can be calculated with Equation (24).


(24) $$P_{a}=\left(M_{a}-M_{b}\right)/M_{a}\times 100\%$$

where $P_{a}$ is the furrow burial return rate, %; $M_{a}$ is the weight of straw in the soil surface layer before ditch burial, g; $M_{b}$ is the weight of straw in the soil surface layer after ditch burial, g.

The data obtained in this study were evaluated using the SPSS 22.0 (SPSS, Chicago, IL, United States) statistical software. Analysis of variance was performed *via* Duncan’s multiple comparison testing with an assessment threshold of *p* < 0.05. The results are expressed as the mean ± standard error. Each repetition was treated as a random effect in the statistical model.

## Results and discussion

### Effect of the experimental factors on the straw picking rate

**Figure 5:**
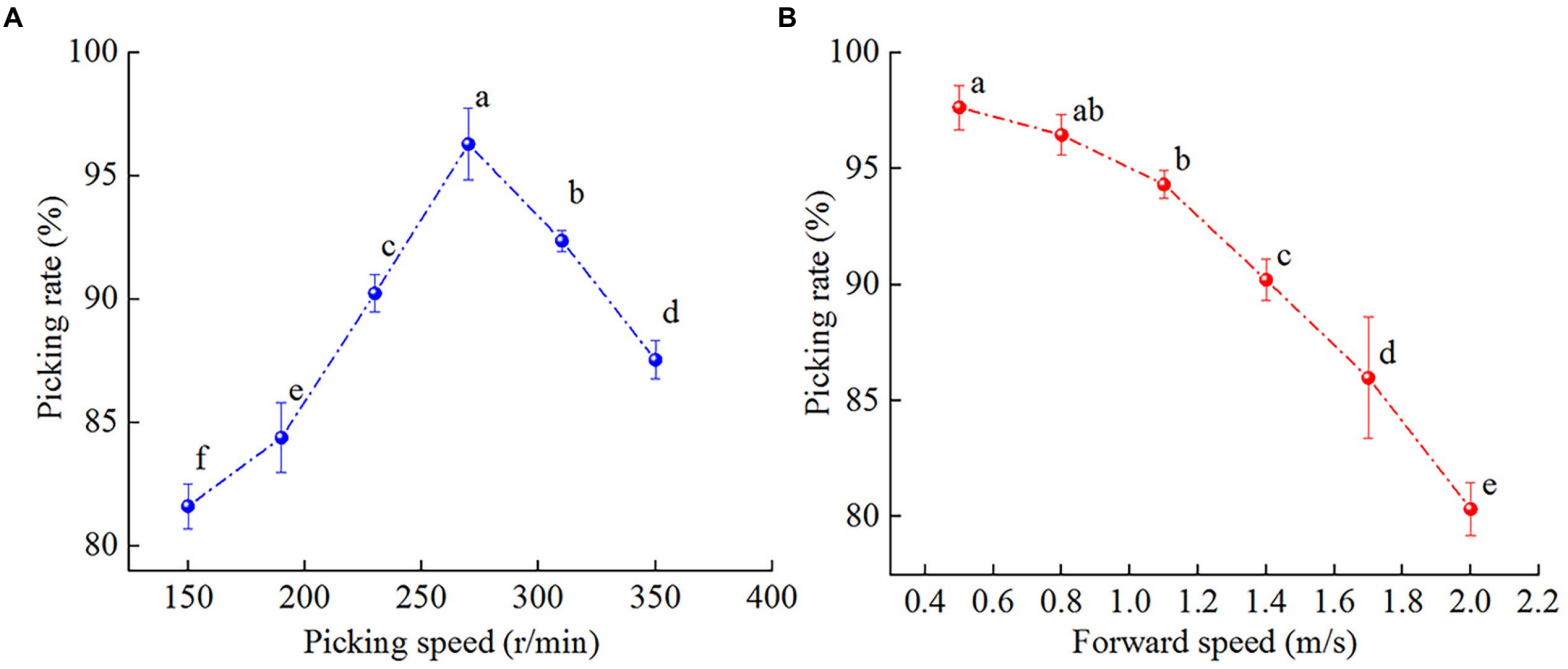
Effect of the experimental factors on the straw picking rate. **(A)** Effect of the forward speed on the straw picking rate; **(B)** effect of the pickup device speed on the straw picking rate. Different letters indicate statistically significant differences (*p* < 0.05).

Figure 5 shows that the forward and rotational speeds of the pickup device significantly affected the straw picking rate (*p* < 0.001). The straw picking rate gradually decreased with increasing forward speed. The main reason was that under the condition of a certain rotational speed of the pickup device, a higher forward speed corresponds to a larger missing picking area formed by the cycloidal motion trajectory interactions between the pickup device and the forward speed and ground. Moreover, the pile phenomenon occurred, so a large amount of straw was missing in the field, which reduced the straw picking rate. The straw picking rate first increased and subsequently decreased with increasing rotational speed of the pickup device. The main reason was that under the condition of a certain forward speed, a higher speed of the pickup device corresponded to more frequently movement trajectory to collect straw per unit time. This effectively reduced the area of the missing picking area. When the speed of the pickup device exceeded 270 r/min, straw was ejected into the carrying area, which made the previously collected straw fall back to the ground and reduced the straw picking rate.

### Effect of the experimental factors on the ditching stability

**Figure 6:**
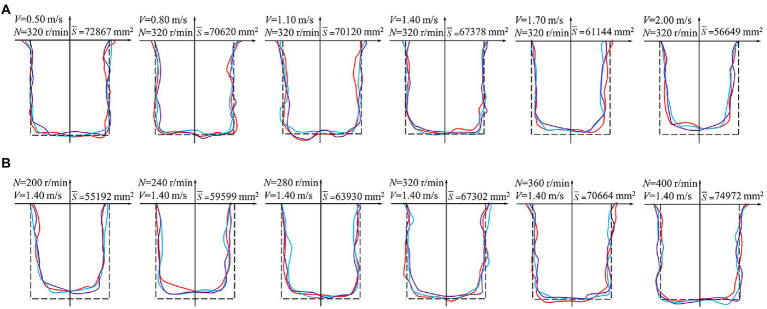
Effect of the experimental factors on the ditch type. **(A)** Effect of the forward speed on the ditch type; **(B)** effect of the ditching device speed on the ditch type. Indicates the theoretical ditch profile. Indicates the first experimental ditch profile. Indicates the second experimental ditch profile. Indicates the third experimental ditch profile.

**Figure 7:**
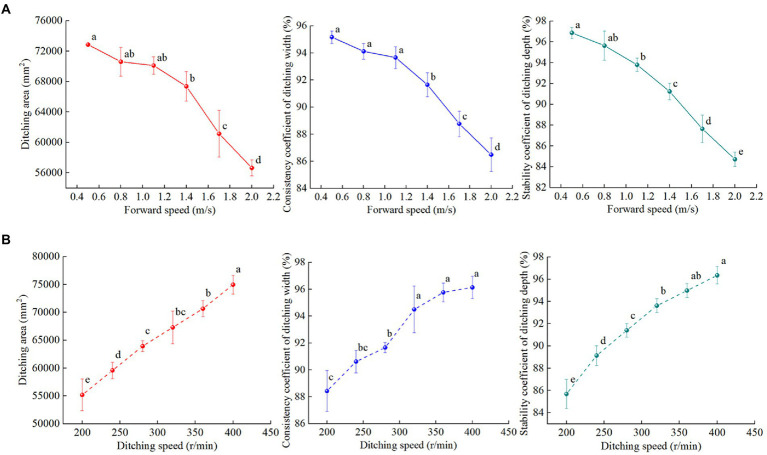
Effect of the experimental factors on the ditching stability. **(A)** Effect of the forward speed on the ditching stability; **(B)** effect of the ditching device speed on the ditching stability. Different letters indicate statistically significant differences (p < 0.05).

Figure 6 shows that under a constant rotational speed of the ditching device, a higher forward speed corresponds to a larger difference between opened and theoretical ditch type profiles. Under a constant forward speed, a higher rotational speed of the ditching device corresponds to a more similar opened ditch type to the theoretical ditch type. To study the influence of the forward and ditching device speeds on the ditching stability, the ditching area, ditching width consistency factor and ditching depth stability factor were separately assessed, as shown in Figure 7.

If the rotational speed of the ditching device remained constant, the ditching area, ditching width consistency factor and ditching depth stability factor were not significant within the range of 0.4–1.0 m/s but were significant within the range of 1.0–2.0 m/s (*p* < 0.001). The ditching area, ditching width consistency factor and ditching depth stability factor gradually decreased with increasing forward speed. The main reason was that a higher forward speed corresponds to a shorter time for the ditching device to impact the soil per unit area, which results in a smaller amount of soil disturbance, a larger difference between opened and ideal ditches, and lower stability of the ditch width and depth.

Under the condition of a constant forward speed, the stability factor of the ditching area and ditching depth significantly influenced the working range of the ditching device speed (p < 0.001). The consistency factor of the ditching area, ditching width and stability factor of the ditching depth gradually increased with increasing speed of the ditching device. This phenomenon largely occurred because a higher rotational speed of the ditching device corresponds to a stronger effect of soil cutting and discharge. As a result, the opened ditch was highly consistent with the contour of the motion track of the ditching knife. The overall structure was relatively flat and closely resembled the ideal ditch type, and the stability of the ditching width and depth was satisfactory.

### Effects of the experimental factors on the straw return rate

Orthogonal rotation combination experiments considering three factors and five levels were conducted with the forward speed, rotational speed of the pickup device and speed of the ditching device as the experimental factors and the straw return rate as the experimental index. The experimental results are summarized in Table 4.

**Table 4:** Experimental results of the orthogonal rotation combination experiments involving three factors and five levels.

Order	Experimental factors			Experimental index
	Forward speed *X*_1_ (m/s)	Picking speed *X*_2_ (r/min)	Ditching speed *X*_3_ (r/min)	Straw return rate *Y* (%)
1	−1 (0.80)	−1 (190)	−1 (240)	92.85
2	1 (1.70)	−1	−1	88.56
3	−1	1 (310)	−1	93.87
4	1	1	−1	92.84
5	−1	−1	1 (360)	95.81
6	1	−1	1	87.06
7	−1	1	1	92.77
8	1	1	1	90.06
9	−1.68 (0.50)	0 (250)	0 (300)	95.78
10	1.68 (2.00)	0	0	89.12
11	0 (1.25)	−1.68 (150)	0	91.84
12	0	1.68 (350)	0	93.49
13	0	0	−1.68 (200)	95.42
14	0	0	1.68 (400)	90.13
15	0	0	0	94.32
16	0	0	0	94.55
17	0	0	0	95.38
18	0	0	0	94.76
19	0	0	0	95.47
20	0	0	0	95.18
21	0	0	0	94.78
22	0	0	0	94.95
23	0	0	0	95.22

The experimental results were analyzed in the ANOVA module based on the Design-Expert 8.0.6 software (Stat-Ease, Inc., Minneapolis, MN, United States). The results are provided in Table 5. The values of the regression terms were significant, and the following regression equation was fitted:


(25) $$\begin{array}{c} Y=94.97-2.05X_{1}+0.59X_{2}-0.83X_{3}+1.16X_{1}X_{2}\\ -0.77X_{1}X_{3}-0.67X_{2}X_{3}-1.04X_{1}^{2}-0.97X_{2}^{2}-0.93X_{3}^{2} \end{array}$$

**Figure 8:**
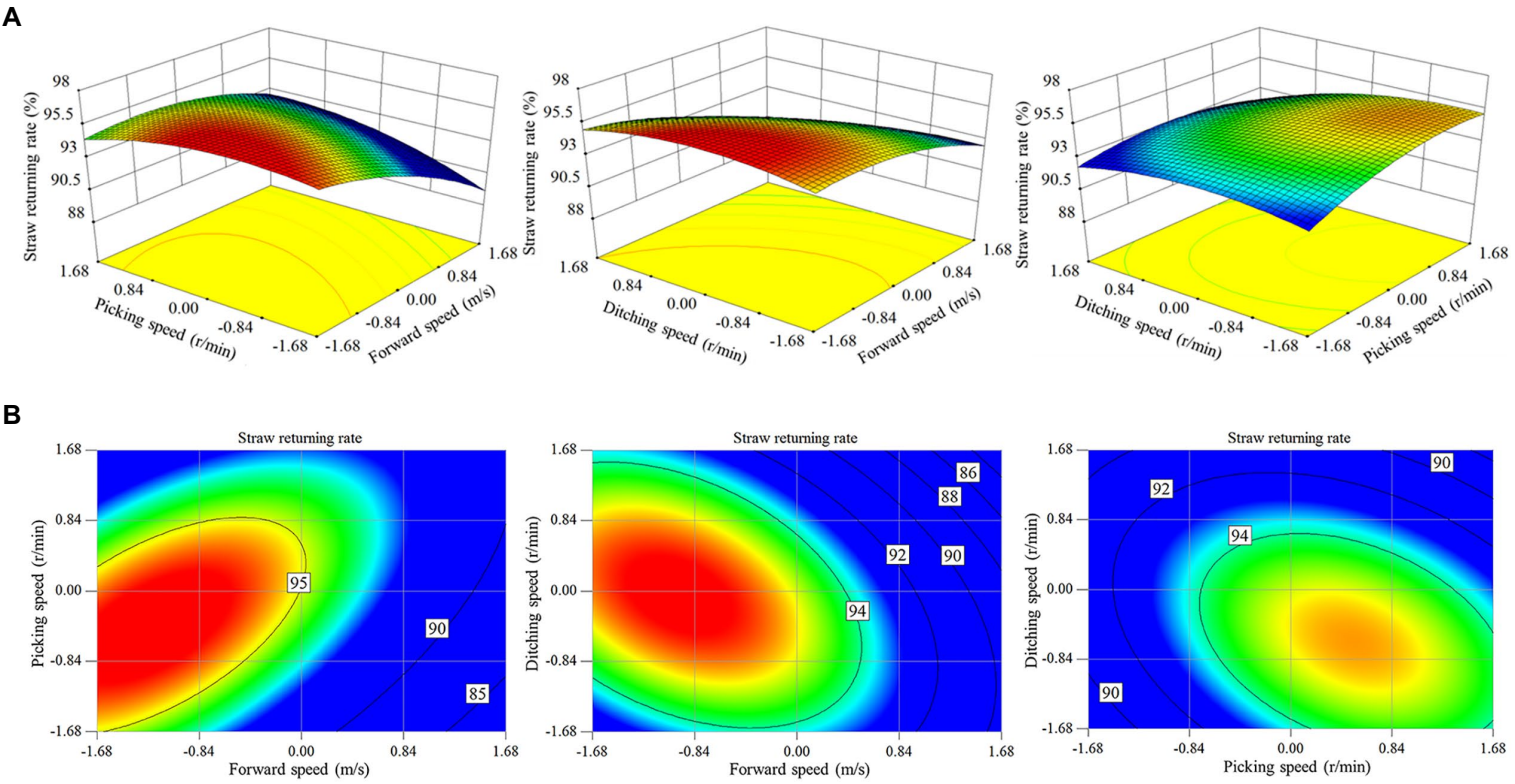
Effect of the experimental factors on the straw return rate. **(A)** Response surface diagram of the interaction among the forward speed, rotational speed of the pickup device and rotational speed of the ditching device; **(B)** contour diagram of the interaction among the forward speed, rotational speed of the pickup device and rotational speed of the ditching device.

According to the *F* value of each experimental factor in the analysis of variance, the primary and secondary factors that affect the straw return rate were *X*_1_ > *X*_1_^2^ > *X*_2_^2^ > *X*_3_^2^ > *X*_1_*X*_2_ > *X*_3_ > *X*_2_ > *X*_1_*X*_3_ > *X*_2_*X*_3_. Within the range of the experimental parameters, the experimental factors that affect the straw return rate significantly differed. To visually describe the effects of the experimental factors and their interactions on the experimental index, response surface and contour plots were generated, as shown in Figure 8.

**Table 5:** Analysis of variance results.

Source	Sum of squares	Degrees of freedom	Mean square	F value	*p* value	Significance
Model	135.83	9	15.09	20.64	<0.0001	^**^
*X*_1_	57.33	1	57.33	78.40	<0.0001	^**^
*X*_2_	4.73	1	4.73	6.46	0.0245	^*^
*X*_3_	9.38	1	9.38	12.82	0.0034	^**^
*X*_1_ *X*_2_	10.81	1	10.81	14.79	0.0020	^**^
*X*_1_ *X*_3_	4.71	1	4.71	6.44	0.0247	^*^
*X*_2_ *X*_3_	3.56	1	3.56	4.87	0.0458	^*^
*X*_1_^2^	17.32	1	17.32	23.69	0.0003	^**^
*X*_2_^2^	14.89	1	14.89	20.37	0.0006	^**^
*X*_3_^2^	13.72	1	13.72	18.76	0.0008	^**^
Residual	9.51	13	0.73			
Lack of fit	8.30	5	1.66	11.05	0.0020	
Pure error	1.20	8	0.15			
Correlation total	145.34	22				

** denotes extreme significance;

* denotes significance.

There was a significant interaction among the forward speed, rotational speed of the pickup device and rotational speed of the ditching device. Under the interaction between forward speed and rotational speed of the pickup device, the straw return rate gradually decreased with increasing forward speed. The straw return rate increased and subsequently decreased with increasing pickup device rotational speed. Under the interaction between forward speed and rotational speed of the ditching device, the straw return rate gradually decreased with increasing forward speed. At a low forward speed, the straw return rate first increased and subsequently decreased with increasing rotational speed of the ditching device. At a high forward speed, the straw return rate gradually decreased with increasing rotational speed of the ditching device. Under the interaction between the rotational speeds of the pickup device and ditching device, the straw return rate gradually increased with increasing rotational speed of the pickup device. The straw return rate first increased and thereafter decreased with increasing rotational speed of the ditching device.

To explore the best parameter combination of various experimental factors and maximize the work efficiency, solution optimization was performed under the condition of maximizing both forward speed and straw return rate. The constraint range of the objective function and working parameters is defined by Equation (26).


(26) $$\{\begin{array}{l} \max Y\left(X_{1},X_{2},X_{3}\right)\\ s.t.\{\begin{array}{l} \max X_{1}\\ 0.50m/s<X_{1}<2.0m/s\\ 150r/\min<X_{2}<350r/\min\\ 200r/\min<X_{3}<400r/\min \end{array} \end{array}$$

The Design Expert 8.0.6 software was employed for optimization. The optimization results were as follows: at a forward speed of 1.68 m/s, the rotational speed of the pickup device was 330 r/min, the rotational speed of the ditching device was 290 r/min, and the straw return rate was 92.82%.

To verify the reliability of the regression model and optimization results, a field verification experiment was conducted considering the best parameter combination (a forward speed of 1.68 m/s, a rotational speed of the pickup device of 330 r/min, and a rotational speed of the ditching device of 290 r/min). The experiment was repeated three times, and the results were averaged. The field experimental results revealed that the straw return rate reached 93.65%. The results of the field validation experiment were consistent with the predicted values of the regression model. The straw return rate satisfies the agronomic requirements under the optimal combination of working parameters.

In this study, the key components of a corn straw ditch-buried returning machine were theoretically analyzed. Through field experiments, the main factors and interactions that affect the rates of picking, ditching and straw return were explored. Traditional straw return is typically accomplished by covering and mixing-based farming. The straw return machine developed in China now achieves a return rate of over 90% (Zhou et al., 2017). This method can mix some straw with soil, but this approach creates certain problems during sowing. Due to the influence of straw, seeds cannot fully contact the soil, which reduces the seed germination rate. To solve these problems, deep burial and return of corn straw are of great importance. The currently developed straw ditch-buried returning machine can bury more than 85% of straw deeper than 100 mm below the ground surface (Zheng et al., 2017; Wang et al., 2020). However, there remain certain problems of a low straw return rate and low depth of return, which affect subsequent planting efforts. In this study, the strip ditch burying and returning farmland agronomic model can effectively avoid these problems. Moreover, the straw return rate was 93.65%. A large amount of straw can be concentrated and deeply buried in soil layers below 300 mm, thereby exceeding the bottom plow layer, which promotes tillage layer thickening.

The straw ditch-buried returning technology breaks the disadvantages of traditional straw returning to the field, which buries all soil in the field. After the crops are mature, only part of the soil is ditched, and the straw is buried. It is the category of less tillage in conservation tillage. This study experimentally examined the effect of straw mulching but did not compare and analyze the changes in organic matter in the soil under the conditions of different straw returning machines, the effects of different straw lengths and different burial depths on the soil. In addition, we did not continue to track the impact on sowing, fertilization, intertillage and harvesting with this background. Kasteel et al. (2007) found that if straw was piled in mounds, it was difficult to achieve adequate decomposition, and the decomposition time in soil increased. In a subsequent study, a decomposing agent spraying device could be added to the rear of the corn straw ditch-buried returning machine. The straw introduced in the ditch could first be sprayed with a decomposing agent and subsequently covered with soil for pressing, which could effectively avoid the impact of chemicals such as humectants on the soil strip in the sowing area. To avoid the impact of straw return to the field on diseases, pests and weeds, herbicides could be sprayed along the ditch, which could help reduce the labor intensity during the middle tillage period. At the later stage, the above mentioned decomposing agent spraying device will be added based on the machines and tools designed in this study. The effects of the decomposition time and decomposing agent on soil moisture and organic matter will be studied. Moreover, the impact of straw on trace elements in soil under this mode will be tracked, and the impact on the entire cycle of crop growth will be explored. The crop growth state will be monitored over a long period. The regulation mechanism of the agronomic model of furrow burial and return, soil and yield will be systematically measured from multiple perspectives and all aspects.

## Conclusion

1. According to the agronomic requirements of corn straw ditch burial and return, a corn straw ditch-buried returning machine that can simultaneously complete the processes of picking, conveying, ditching, covering soil and pressing was designed. Key components were designed, such as the pickup device, chain ditching device and straw-guiding soil-covering and pressing device.
2. The straw picking rate gradually decreased with increasing forward speed and first increased and subsequently decreased with increasing rotational speed of the pickup device. The ditching stability decreased with increasing forward speed and increased with increasing rotational speed of the ditching device. The forward and rotational speeds of the pickup and ditching devices significantly affected the straw return rate. At a forward speed of 1.68 m/s, the rotational speed of the pickup device was 330 r/min, the rotational speed of the ditching device was 290 r/min, and the straw return rate was 93.65%.
3. This study combined theory and experiments to effectively improve the rate of corn straw return. This study provides references for the innovative design of straw returning equipment and continued exploration of ideas of conservation tillage modes.

## Data availability statement

The original contributions presented in the study are included in the article/supplementary material, further inquiries can be directed to the corresponding author.

## Author contributions

HT, CX, WX, YX, and JW designed and performed the experiments and analyzed the data. HT, CX, and YSX wrote the manuscript. All authors contributed to the article and approved the submitted version.

## Funding

This work was supported by the National Natural Science Foundation of China (NSFC), grant number: 32071910; the Program on Industrial Technology System of National Rice (CN), grant number: CARS-01-48.

## Conflict of interest

The authors declare that the research was conducted in the absence of any commercial or financial relationships that could be construed as a potential conflict of interest.

## Publisher’s note

All claims expressed in this article are solely those of the authors and do not necessarily represent those of their affiliated organizations, or those of the publisher, the editors and the reviewers. Any product that may be evaluated in this article, or claim that may be made by its manufacturer, is not guaranteed or endorsed by the publisher.
